# Timely initiation of breastfeeding and its association with birth place in Ethiopia: a systematic review and meta-analysis

**DOI:** 10.1186/s13006-017-0133-x

**Published:** 2017-10-03

**Authors:** Animut Alebel, Getiye Dejenu, Getachew Mullu, Nurilign Abebe, Tenaw Gualu, Setegn Eshetie

**Affiliations:** 1 College of Health Sciences, Debre Markos University, P.o.box: 269, Debre Markos, Ethiopia; 2 College of Medicine and Health Sciences, University of Gondar, Gondar, Ethiopia

**Keywords:** Prevalence, Timely initiation, Early initiation, Breastfeeding, Place of birth, Ethiopia

## Abstract

**Background:**

Timely initiation of breastfeeding is defined as putting the newborn to the breast within 1 h of birth. In Ethiopia, different studies have been conducted to assess the prevalence of timely initiation of breastfeeding and associated factors. The findings of these studies were inconsistent and characterized by great variability. Therefore, the aim of this systematic review and meta-analysis was to estimate the pooled prevalence of timely initiation of breastfeeding and its association with birth place in Ethiopia using the available studies.

**Methods:**

Databases, including PubMed, Google scholar, Science direct and Cochrane library were systematically searched. All original studies reporting the prevalence of timely initiation of breastfeeding in Ethiopia were considered. Two authors independently extracted all necessary data using a standardized data extraction format. STATA 11 statistical software was used to analyze the data. The Cochrane Q test statistics and *I* ^*2*^ test were used to assess the heterogeneity between the studies. A random effect model was computed to estimate the pooled prevalence of timely initiation of breastfeeding. In addition, the associations between timely initiation of breastfeeding and place of birth were determined.

**Results:**

Sixteen studies were finally included in the meta-analysis. The findings of this meta-analysis revealed that, the pooled prevalence of timely initiation of breastfeeding in Ethiopia was 61.4% (CI: 51.4, 71.5%). The study also indicated that rural mothers had lower rate of initiating breastfeeding within the first 1 h after delivery as compared to their urban counterparts. Additionally, mothers who gave birth at health institution were almost 2.11 times more likely to initiate breastfeeding within 1 h as compared to mothers who did not give birth at health institution.

**Conclusion:**

In this study, timely initiation of breastfeeding in Ethiopia was significantly low compared to the current global recommendation on breastfeeding. Women from rural area were less likely to initiate breastfeeding within 1 h as compared with women from urban areas. Mothers who give birth at health institution were more likely to initiate breastfeeding timely.

## Background

Timely initiation of breastfeeding is defined as putting the newborn to the breast within 1 h of birth [1]. Breast milk is the best food for the infant for the first 6 months of life and it should be initiated within 1 h of birth, even before the expulsion of the placenta [2]. Some of the advantages of breastfeeding include; it increases bonding between mother and infant, reduces the risk of upper respiratory tract infections in the child, enhances dental development, aids in cognitive development, and decreases the risk for obesity in later life [3].

Timely initiation of breastfeeding has a major contribution to the survival of neonate. The study showed that 22% of neonatal mortality could be prevented if breastfeeding was initiated within the first hour [4]. Despite of this fact only (45%) of newborns started breastfeeding within the first hour of life globally. The result is even lower in developing countries. For example, West and Central Africa (40%), East Asia and Pacific (44%), South Asia (42%), Latin America and Caribbean (49%), Eastern and southern Africa (59%) of newborns initiated breastfeeding within an hour of delivery [5]. In Ethiopia, breastfeeding practice is universal where more than 90% of mothers breastfeed, but it is sub optimal [6, 7].

Late initiation of breastfeeding is associated with morbidity and mortality of newborns. There was a marked dose response of increasing risk of neonatal mortality with increasing delay in initiation of breastfeeding from 1 h to day 7; overall late initiation (after day 1) was associated with a 2.4-fold increase in risk of death [4]. Providing appropriate breastfeeding in the first day of life is crucial to the health of the newborn infant and to breastfeeding success [8, 9]. Progress to improve early initiation rates has been slow over the past 15 years; with global rates increasing by just 14 percentages point overall. Still less than half of all newborns are put to the breast within an hour of birth globally [5].

In Ethiopia, different studies have been conducted to assess the timely initiation of breastfeeding and associated factors [10–25]. The findings of these studies were inconsistent and characterized by great variability. Several factors were also identified, and place of birth and mode of delivery were the most frequently mentioned factors [12, 13, 16, 18]. Other factors include; having information about breastfeeding [12, 14, 20], colostrum feeding [12, 23], maternal education [16, 24], antenatal care [11, 25] and postpartum counseling about breastfeeding [14].

These studies presented controversial findings concerning the association between place of birth and timely initiation of breastfeeding. In some studies, place of birth was positively associated with timely initiation of breastfeeding, while other studies showed negative association. Such discrepancy is not well investigated. Therefore, the aim of this meta-analysis was to estimate the pooled prevalence of timely initiation of breastfeeding and its association with place of birth in Ethiopia. The findings of this study will be an input to policy makers and program planners in the design of appropriate interventions to improve early breastfeeding initiation. The study will have a paramount importance for clinicians and future researchers in related topics.

## Methods

### Searching strategies

To ensure scientific rigor, the Preferred Reporting of Systematic Reviews and Meta-Analysis (PRISMA) guidelines were used [26]. The current systematic review and meta-analysis was conducted based on the review of different literatures. The international databases, including PubMed, Google scholar, Science direct and Cochrane library were systematically searched. The search was carried out using the following keywords “prevalence”, “timely initiation of breastfeeding”, “Early initiation of breastfeeding”, “institutional delivery”, skilled birth attendants, and “Ethiopia”. The search terms were used separately and in combination using Boolean operators like “OR” or “AND”. The search was conducted from the 1st of January to the 28th of February, 2017. All papers published until the 28th of February, 2017 were included in the review.

### Eligibility criteria

#### Inclusion criteria

**Study area**: Only studies conducted in Ethiopia.

**Publication condition:** articles published in peer reviewed journals.

**Study design:** all original studies report the prevalence of timely initiation (initiation of breastfeeding within 1 h of delivery) of breastfeeding and its associations with place of birth were considered.

**Language:** Articles published in English language.

#### Exclusion criteria

We excluded article, which were not fully accessed, after we contact the primary author two times through email. We exclude these articles because of we were unable to assess the quality of each article without accessing the full text.

### Outcome measurement

The primary outcome of this study was the timely initiation of breastfeeding. The prevalence was calculated by dividing the number of women initiating breastfeeding within 1 h to the total number of women who have ever breastfed. The second outcome of the study was to determine the association between timely of breastfeeding and institutional delivery.

### Data abstraction

Two authors independently extracted all necessary data using a standardized data extraction format. The data extraction format includes primary author, publication year, region of the study (study site in the country), sample size, response rate and prevalence of timely initiation of breastfeeding.

### Quality assessment

Newcastle-Ottawa Scale adapted for cross-sectional studies quality assessment tool was used to assess the quality of each study [27]. The tool has mainly three sections the first section graded from five stars and mainly focused on the methodological quality of each study. The second section of the tool deals with the comparability of the study. The last section deals with the outcome and statistical analysis of each original study. Two authors independently assessed the quality of each original study using the tool. Moreover, disagreements between two authors were resolved by taking the mean score of the two authors. Finally, research’s with a scale of ≥6 out of 10 scales were considered as high quality.

### Statistical analysis

Data analysis was carried out by using STATA 11 statistical software. The standard error for each original study was calculated using the binomial distribution formula. Heterogeneity among reported prevalence was assessed by computing values for chi-square, I^2^ and *p*-values [28]. As the test statistic showed there is a significant heterogeneity among studies (I^2^ = 99.4%, *p* = 0.00) as a result random Effects model was used to estimate the Der Simonian and Laird’s pooled effect. To minimize the random variations between the point estimates of the primary study subgroup analysis was done based on the type of population included under each study (urban and rural). In addition, to identify possible source of heterogeneity Univariate Meta regression was undertaken by taking the sample size and year of publication but none of them were statistically significant. Furthermore, Egger and Begg tests at 5% significant level were employed for assessing publication bias [29]. Point prevalence as well as their 95% confidence interval were presented in forest plot. In this plot, the size of each box indicated the weight of the study, while each crossed line refers to 95% confidence interval. For the second outcome Logs odds ratio was used to determine the association between timely initiation of breastfeeding and institutional delivery.

## Results

In the first step of our search, 360 articles were retrieved regarding timely initiation of breastfeeding through PubMed, Google scholar, science direct and others. Of which, 115 were excluded due to duplicated articles. From the remaining 245 articles, 162 articles were excluded after reviewing of their titles and abstracts because their titles were found to be irrelevant. The rest 83 articles were screened for full text and 59 were excluded due to the outcome of interest. Therefore, 24 full text articles were accessed and assessed for eligibility based on the pre-set criteria. Finally, 16 studies were fulfilled the eligibility criteria and included in the meta-analysis.

**Fig. 1:**
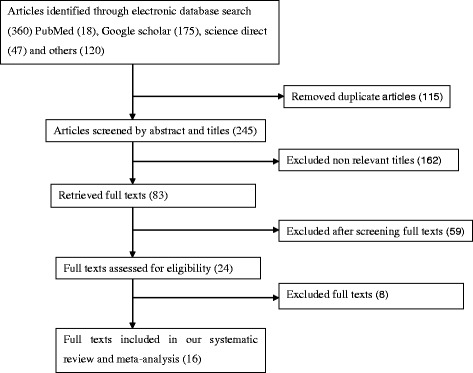
Flow chart to describe the selection of studies for a systematic review and meta-analysis of the prevalence of timely initiation of breastfeeding and is association with birth place in Ethiopia

From 24 full articles accessed eight were excluded because they were conducted in other developing countries: Ghana [30], Nigeria [31], Tanzania [32], Uganda [33, 34], India [24, 35] and in three African countries (Burkina Faso, Uganda and South Africa) [36] (Fig. 1).

### Characteristics of original studies

As described in Table 1, these 16 studies were published from 2011 to 2017. In the current meta-analysis, 18,870 breastfeeding women’s were involved to estimate the pooled prevalence of timely initiation of breastfeeding. Regarding study design, all studies are cross sectional study design. The highest prevalence (88.5%) of timely initiation of breastfeeding was reported from a study done in Nekemte town, Oromia regional state [11] and the lowest prevalence (13.8%) was reported from a study done in Black Lion Hospital, Addis Ababa [19]. In this meta-analysis, six regions of the country were represented. Two of the studies were from Tigray region [22, 23]; five studies were from Amhara region [12, 13, 15, 17, 25], one from Addis Ababa [19], four from Oromia region [10, 11, 14, 20], two from Southern Nations, Nationalities and peoples’ region [21, 24] (SNNPR), one from Afar region [16] and one nationwide study [18]. About half (eight) of the studies were conducted both in urban and rural settings [10, 14, 16–19, 21, 22]. Five studies were conducted in urban settings [11–13, 15, 23] and the rest three studies were conducted in rural settings [20, 24, 25]. Moreover, the response rate of each original study ranged from 95.7 to 100%, almost all studies had a good response rate. This high response rate could be resulted from most of the studies used interviewer administer questionnaire to collect the data (Table 1).

**Table 1:** Descriptive summary of 16 studies included in the meta-analysis of the prevalence of timely initiation of breastfeeding in Ethiopia, 2017

Region	Study area	Authors name	Publication year	Response rate (%)	Sample size	Prevalence (95% CI)
Tigray	Axum town	Alemayehu et al. [23]	2014	100	418	41.6 (36.9, 46.4)
	Mekelle town	Berhe et al. [22]	2013	100	361	77 (72.7, 81.3)
Amhara	Dembecha district	Bimerew et al. [25]	2016	100	739	73.1 (69.9, 76.3)
	Motta town	Tewabe [13]	2016	95.7	405	79 (75, 83)
	Debre Berhan town	Tilahun et al. [12]	2016	98.3	409	62.6 (57.9, 67.3)
	Raya Kobo district	Legesse et al. [17]	2014	99.5	623	71.7 (68.2, 75.3)
	Bahir Dar City	Abdulbasit Musa [15]	2014	100	815	87 (84.7, 89.3)
Addis Ababa	Black Lion Hospital	Hailu Berta Deregh [19]	2012	100	429	13.8 (10.5, 17)
Oromia	Goba district	Setegn et al. [14]	2011	99.01	599	52.4 (48.4, 56.4)
	Nekemte town	Wolde et al. [11]	2014	96	174	88.5 (83.5, 93.2)
	East Wollega zone	Hailemariam et al. [20]	2015	99.8	593	83.1 (80.1, 86.2)
	Tiyo Woreda	Bedasa W/Michael [10]	2016	96.6	373	32.7 (27.9, 37.5)
Southern Nations Nationalities and peoples’ region	Arba Minch Zuria	Adugna [24]	2014	99.7	383	42.8 (37.9, 47.8)
	Dale Woreda	Beyene et al. [21]	2017	100	617	83.8 (80.9, 86.7)
Afar	Amibara district	Liben and Yesuf [16]	2016	99	380	39.7 (34.8, 44.7)
EDHS 2011 based	EDHS 2011 based	Lakew et al. [18]	2015	__________	11,552	53.5 (52.5, 54.4)

### Meta-analysis

**Fig. 2:**
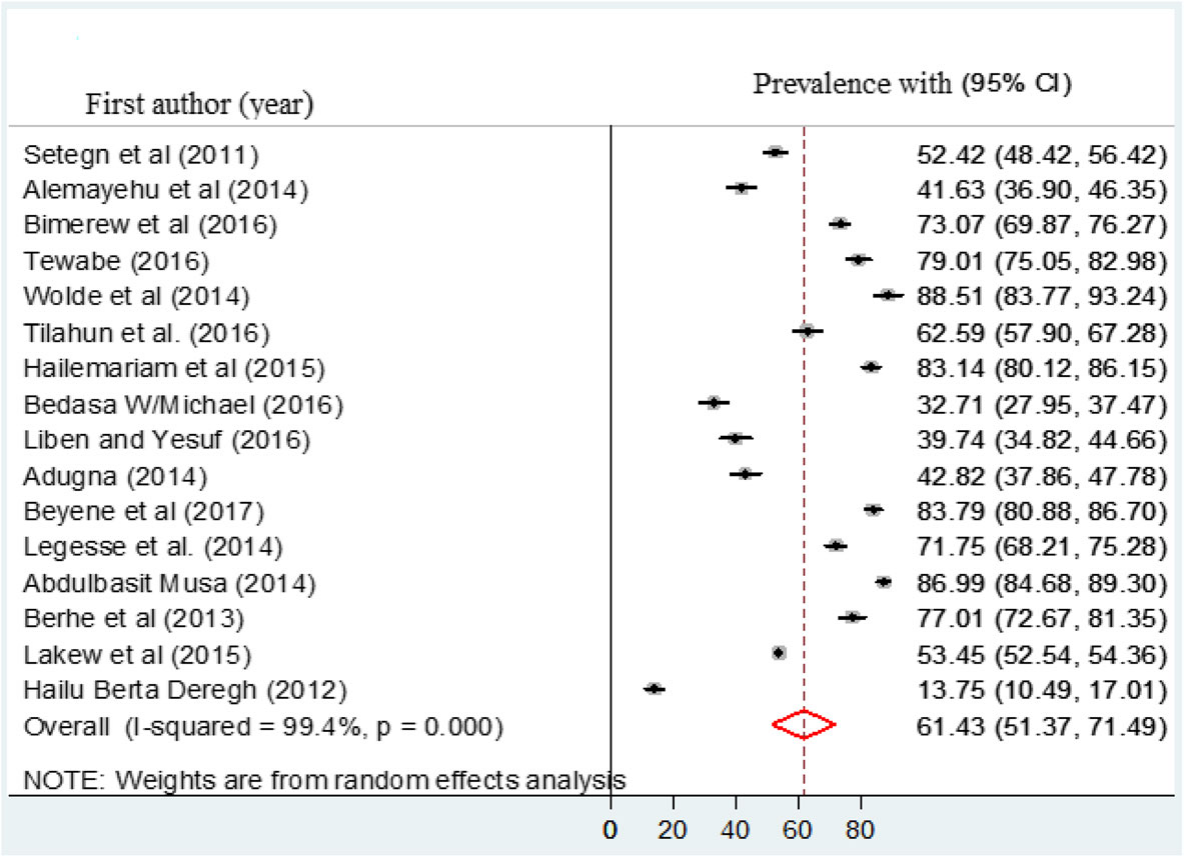
Forest plot of the pooled prevalence of timely initiation of breastfeeding in Ethiopia

The result of 16 studies revealed that the pooled prevalence of timely initiation of breastfeeding in Ethiopia was 61.4% (CI: 51.4, 71.5%) (Fig. 2). Severe heterogeneity was observed across the studies (I^2^ = 99.4, *p* value = 0.000). As a result, a random effect model was employed to estimate the pooled prevalence of timely initiation of breastfeeding. Different factors associated with the heterogeneity such as publication date and sample size of the study were investigated using univariate meta-regression models, and none of these variables were statistically significant (Table 2). Publication bias was also assessed using Egger and Begg tests. The result of Begg and Eegger tests were not statistically significant for estimating the prevalence of timely initiation of breastfeeding (*p* = 0.07) and (*p* = 0.5) respectively.

**Table 2:** Related factors with heterogeneity of timely initiation of breastfeeding prevalence in the Current meta-analysis (Based on Univariate Meta Regression)

Variables	Coefficient	*P*-value
Publication year	0.037	0.262
Sample size	−0.000	0.685

### Subgroup analysis

In addition, in this meta-analysis, we preformed subgroup analysis based on the geographical setting (Region of the country) of the studies. Accordingly the highest prevalence was observed in Amhara region with prevalence of 74.8% (95% CI: 66.5, 83.1%) followed by Oromia region with prevalence of 64.2% (95%CI: 39.9, 88.6%) (Table 3). Besides, to this we also performed subgroup analysis based on residence (urban and rural) of study participants. From the result of this subgroup analysis, we found that urban women’s, 70.9% (95% CI: 61.1, 80.8%) had a higher prevalence of timely initiation of breastfeeding than rural women’s 50.1% (95% CI: 33.3, 66.8%) (Table 3).

**Table 3:** Subgroup prevalence of timely initiation of breastfeeding in Ethiopia, 2017 (*n* = 16)

Variables	Characteristics	Estimate (95% CI)	*P*-value
By region	Oromia	64.2 (39.9, 88.6)	< 0.001
	Amhara	74.8 (66.5, 83.1)	
	Others	50.3 (34.5, 66.2)	
By study setting	Urban	70.9 (61.1, 80.8)	< 0.001
	Rural	50.1 (33.3, 66.8)	
	Overall	61.4 (51.4, 71.5)	

### The association between place of birth and timely initiation of breastfeeding

**Fig. 3:**
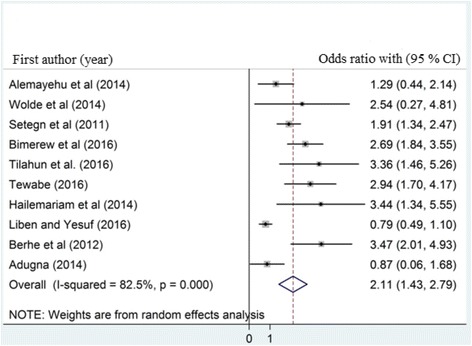
The pooled odds ratio of the association between timely initiation of breastfeeding and place of birth

A total of 10 studies which examined the association between timely initiations of breastfeeding and place of birth were included (Table 4). The analysis of ten studies revealed that institutional delivery was strongly associated with timely initiation of breastfeeding OR 2.11 (95% CI: 1.43, 2.79). The result of this meta-analysis indicated that mothers who gave birth at the health institution were 2.11 times more likely to initiate breastfeeding within 1 h as compared to mothers who gave birth at Home (Fig. 3). We also carried out sensitivity analysis and statistically significant difference was detected for two studies as a result, two studies were excluded [10, 21].

**Table 4:** Individual study data to calculate the odds ratio of timely initiation of breastfeeding and its association with place of birth in Ethiopia, 2017

Sr/no	Authors	Publication year	Institutional delivery	Initiation of breastfeeding (< 1 h)		OR
				Yes	No	
1.	Alemayehu et al. [23]	2014	Yes	161	13	1.289
			No	221	23	
2.	Wolde et al. [11]	2014	Yes	145	9	2.544
			No	19	3	
3.	Setegn et al. [14]	2011	Yes	106	202	1.907
			No	60	218	
4.	Bimerew et al. [25]	2016	Yes	470	70	2.695
			No	142	57	
5.	Tilahun et al. [12]	2016	Yes	238	18	3.36
			No	122	31	
6.	Tewabe [13]	2016	Yes	278	41	2.938
			No	60	26	
7.	Hailemariam et al. [20]	2014	Yes	241	15	3.443
			No	126	27	
8.	Liben and Yesuf [16]	2016	Yes	85	66	0.793
			No	143	88	
9.	Berhe et al. [22]	2012	Yes	237	41	3.468
			No	50	30	
10.	Adugna [24]	2014	Yes	7	212	0.869
			No	6	158	
Overall OR						2.11

### The time trend of timely initiation of breastfeeding in Ethiopia

**Fig. 4:**
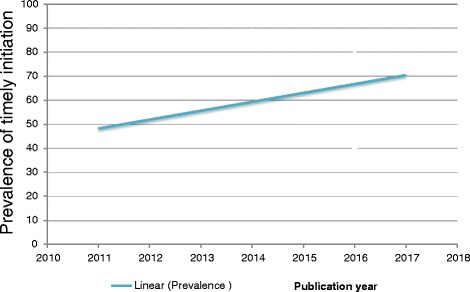
The time trend of timely initiation of breastfeeding in Ethiopia from 2011 to 2017

Our study also describes the time trend of timely initiation of breastfeeding in Ethiopia from 2011 to 2016. In this finding, the general linear trend of timely initiation of breastfeeding was increased in each successive year (Fig. 4).

## Discussion

This meta-analysis was aimed to estimate the pooled prevalence of timely initiation of breastfeeding and its association with birthplace. Although the WHO [37] and Ethiopian IYCF [38] guidelines recommends that all newborns should start breastfeeding immediately after birth, the present meta-analysis reported that only 61.4% (95% CI: 51.4, 71.5%) of newborn were benefited from early initiation of breastfeeding in Ethiopia. According to WHO infant and young children feeding rate on early initiation of breastfeeding, 0–29% considered as poor, 30–49% considered as fair, 50–89% considered as good and 90–100% considered as very good [37]. Therefore, the findig of this study showed that the prevalence of timely initiation of breastfeeding in Ethiopia was gnerally good (61.4%). However, it was lower than Ethiopian HSDP IV target level. The target planned to increase the proportion of timely initiation breastfeeding mother from 69 to 92% by the end of 2015 [39]. The finding of this study indicated that even Though the Ethiopian government has implemented different strategies to improve early initiation of breastfeeding but the present study underscores a lot needed to accomplish target levels.

The prevalence noted in this study was higher than that reported in Pakistan (29%), India (41%), Bangladesh (47%) and Nepal (45%) [40]. Similarly, the finding of this study was also higher than other sub-Saharan countries Demographic and Health survey (DHS based studies: Nigeria (47%) [31] and Tanzania (52%) [32]. The possible explanation for higher rate of early initiation of breastfeeding in this study could be due to the fact that methodological difference, variation in infant and maternal socio-demographic characteristics, economical and health service utilization.

The subgroup analysis of this study revealed that there is a significant variation among Ethiopian regional states in the prevalence of timely breastfeeding initiation. Mothers who were from Amhara region had higher rates of timely initiation of breastfeeding as compared to mothers from Oromia and other regions. The possible source of variation in the regional prevalence could be due to difference in the implementation of health extension programs. Besides, it is also noted that rural mothers had lower rate of timely initiation of breastfeeding as compared to their urban counterparts. This finding is consistent with the 2011 Ethiopian Demographic and Health Survey report. The Ethiopian DHS report indicated that rural women had lower prevalence of timely initiation of breastfeeding than urban [41]. This could be due to the difference in traditional beliefs and cultural practices among mothers in Ethiopia. The discarding of colostrum is a common practice among rural Ethiopian mothers. Mothers who discard the colostrum may take more than 1 h to discard it and therefore initiation of breastfeeding might be late [17, 18]. The other possible explanation might be due to mothers who live in urban places might have a good access to different information sources on breastfeeding including media. On the other hand, rural mothers might not have access to such information sources. Different earlier studies which have been conducted in Ethiopia reported that mothers who had access to mass media like radio or television were more likely to initiate breastfeeding within 1 h than their counterparts [20, 25]. Moreover, this variation might be resulting from due to lack of knowledge on the right time of breastfeeding initiation [14].

The other aim of the study was to determine the association between timely initiation of breastfeeding and place of birth. Accordingly, place of birth was significantly associated with timely initiation of breastfeeding. Women’s who gave birth at health institution were almost 2.11 times more likely to initiate breastfeeding within 1 h as compared to women who had birth other than health institution. This is consistent with studies which have been conducted in Nepal [42] and Tanzania [32]. This could be due to the effect of postpartum counseling on the importance of timely breastfeeding initiation for women who delivered at health facility. The other possible explanation could be due to mothers who gave birth at home in Ethiopian communities were more commonly practiced prelacteal feeding to the new born prior initiation of breastfeeding this may results late initiation of breastfeeding. There are supportive evidences from Ethiopia which revealed that mothers who gave birth at home were more likely to practiced prelacteal feeding than their counterparts [17, 43].

Lastly, this study also describes the time trend of timely initiation of breastfeeding in Ethiopia for the past 8 years. It is observed that the general linear trend of timely initiation of breastfeeding was slightly increased from 2011 to 2017. The possible reason might be due to the Federal government has developed infant and young child feeding guidelines giving appropriate emphasis to key messages on timely initiation of breastfeeding since 2004 [44]. Additionaly, different interventions are in place as breastfeeding promotions have been given at health institutions and at the community level by community health extension workers and other health care providers.

### Limitations of the study

The first limitations of the study was only English articles or reports were considered to carried out the analysis. All studies included studies in this review were cross sectional in nature and therefore the outcome variable might be affected by other confounding variables. The limited sample size could affect the estimated report. Moreover, this meta-analysis represented only studies reported from six regions of the country and one study from EDHS based.

## Conclusion

The pooled prevalence of timely initiation of breastfeeding in Ethiopia was significantly low as compared to the global recommendation on breastfeeding. Women who lived in urban area initiate breastfeeding early as compared to women in rural area. Place of birth has a significant impact on timely initiation of breastfeeding. Therefore, health care workers (midwives and obstetricians) should focus on increasing timely initiation of breastfeeding by giving postnatal counseling regarding to the importance of early initiation of breastfeeding. Moreover, a special emphasis should be given for rural women and women who gave birth at home to increase timely initiation of breastfeeding.

## Abbreviations

DHS: Demographic and Health survey
EDHS: Ethiopian Demographic and Health survey
HSDP: Health Sector Development Plan
WHO: World Health Organization
